# A biopsychosocial approach to sleep health during puberty: Individual and contextual aspects and the role of gender differences. A narrative review

**DOI:** 10.1080/28324765.2025.2541697

**Published:** 2025-08-07

**Authors:** Debora Meneo, Elisabetta Baldi, Fee Benz, Matteo A. Fabris

**Affiliations:** a Department of Human Sciences, Guglielmo Marconi University, Rome, Italy; b Department of Psychiatry and Psychotherapy, Medical Center - University of Freiburg, Freiburg, Germany; c Department of Psychology, University of Turin, Turin, Italy

**Keywords:** puberty, sleep health, gender differences, psychological health, development

## Abstract

Puberty is a period of rapid physical, psychological, and social development marked by significant hormonal shifts and brain reorganization. These changes influence various aspects of early adolescents’ lives, including sleep patterns. Sleep is essential for healthy development, impacting cognitive functions, psychological well-being, and physical health. During puberty, sleep-wake regulation undergoes substantial modifications, often leading to changes in subjective sleep quality, daytime alertness, sleep timing, efficiency, duration, and sleep-related behaviours. This review aims to examine the intricacies of sleep during puberty, taking a biopsychosocial approach to the factors at different levels that affect sleep health changes, including individual-level (hormonal, neurocognitive, and psychological), contextual-level (school, family, peers, and social media use), and cultural and environmental-level (social roles, socio-economic factors and cultural aspects), with a focus on the differences observed between boys and girls. Clinical implications for promoting sleep health and treating sleep difficulties in puberty will be discussed. Understanding the different factors affecting sleep health in puberty and the sex-specific sleep patterns is crucial for addressing the unique needs of pubertal girls and boys and promoting their overall well-being.

## Introduction

The onset of adolescence, marked by puberty, brings a plethora of biological, psychological and social changes that lead to new health concerns, including psychological difficulties, at-risk behaviours and sleep health challenges (Lucien et al., 2021; Mendle, 2014; Pfeifer & Allen, 2021). Most notably, the transformations occurring during puberty lead to: rapid physical development, with the emergence of secondary sex characteristics and changes in physical appearance; increased peer and school stress, symptoms of anxiety and depression, at risk behaviours, and difficulties with self-esteem; changes in family dynamics, with shifts in parent–child relationship (e.g., less parental control, more conflicts) mainly due to early adolescents’ increased strive for independence and reliance on peers relationships (Pfeifer & Allen, 2021; Suleiman & Dahl, 2019).

Puberty and adolescence are often used as almost overlapping terms. However, it is critical to use precise definitions to understand how these phases influence the brain’s response to hormones (Holder & Blaustein, 2014) and the person’s adjustment to physical, psychological, and social changes (Pfeifer & Allen, 2021). Puberty is a prolonged developmental process, describing the transition from a non-reproductive state to reproductive competence (Sisk & Foster, 2004; Waylen & Wolke, 2004). As such, puberty is marked by hormonal changes, including the development of secondary sexual characteristics, depending on oestrogens and progestins in girls and androgens in boys (Marshall & Tanner, 1969, 1970; Tanner, 1962). Adolescence refers to the social and cognitive maturation associated with and resulting from the hormonal changes of puberty (Sisk & Foster, 2004). The hallmarks of adolescence are less clearly defined than those of puberty and include the development of the cortical and limbic areas of the brain (Holder & Blaustein, 2014).

Pubertal development is often classified based on Tanner Staging (Tanner, 1962). This classification tracks the development and sequence of secondary sex characteristics during puberty (pubic hair for both sexes, breast for girls, and testicular volume for boys): from Tanner Stage 1, corresponding to pre-pubertal form, to Tanner Stage 5, corresponding to adult form. Pubertal development is initiated by changes in the hypothalamic-pituitary-gonadal (HPG) axis, with consequent changes in various hormonal systems (Mendle et al., 2019). These trigger modifications in physical appearance, social environment, and brain functioning, affecting boys and girls on cognitive, emotional, and behavioural levels (R. E. Dahl et al., 2018). Typically, girls begin pubertal development around ages 10–11 and complete the process by ages 15–16, whereas boys begin puberty slightly later at ages 11–12 and complete it by 16–17 (Marshall & Tanner, 1969; Tanner, 1962). However, a secular trend has been observed in earlier pubertal onset in girls (Eckert-Lind et al., 2020).

The changes associated with pubertal development are posited to start a cascade of events, from brain maturation to socio-affective adaptation, happening in adolescence (Berenbaum et al., 2015; Blakemore et al., 2010; Pfeifer & Allen, 2021). One crucial change occurring with pubertal development is in sleep-wake regulation (S. J. Crowley et al., 2018; Rapee et al., 2019). These changes involve different aspects of sleep, broadly defined as sleep health. Sleep health as a multidimensional construct defined by positive attributes was proposed by Daniel Buysse in 2014 and adapted to the developmental age by Lisa Meltzer and colleagues in 2021. Six core dimensions of sleep health have been identified by Meltzer et al. (2021) for the paediatric population: sleep satisfaction (the subjective assessment of one’s own sleep quality); alertness during the day (the ability to sustain attentive wakefulness during the day, as opposed to daytime sleepiness); sleep timing (the allocation of the main sleep episode in the 24-hour day); sleep efficiency (or continuity, i.e. the ability to fall asleep easily and to remain asleep until the desired waketime); sleep duration (the amount of sleep in the 24 h); sleep-related behaviours (pre-sleep activities compatible with sleep, consistent bedtime routines, and regularity of sleep habits). Each of these dimensions is associated with healthy development and can be independently assessed subjectively and/or physiologically (Meltzer et al., 2021).

This framework emphasizes the positive attributes of sleep and aims to promote healthy sleep. Rather than pathologizing physiological aspects of sleep in different age groups, it seeks targets for improving sleep based on age and individual differences. Indeed, sleep health is posited to be a continuum: on one side, good sleep health is defined by a positive state of the five dimensions (six in the developmental age); on the opposite side, poor sleep health can include the presence of a sleep-wake disorder (Buysse, 2014). For instance, poor sleep efficiency can be configured as a persistent difficulty in initiating or maintaining sleep, i.e. insomnia disorder, which become more prevalent after puberty (e.g., Zambotti et al., 2017). The puberty-related increase in insomnia is paralleled by an increase in other psychopathology (Graber, 2013; J. Zhang et al., 2016), especially in girls (Suh et al., 2018). Insomnia disorder is longitudinally associated with an increased risk for mental health problems during adolescence (Uccella et al., 2023). Moreover, insomnia and other sleep difficulties tend to persist from childhood to adolescence and to aggravate during puberty (Roberts et al., 2008; Wang et al., 2016).

Research attention to insomnia and its correlated health risks in adolescents has increased in the last decades (Gao et al., 2023), testifying to the growing recognition of sleep as a key aspect of healthy development in teens. It is of utmost importance to gain a better understanding of sleep health in this population, both to prevent long-lasting negative outcomes of sleep difficulties and to promote sleep health as a crucial aspect of global health (Buysse, 2014) and healthy development (Meltzer et al., 2021).

Adopting the framework of sleep health is pivotal to understanding the changes in sleep during puberty. This can provide insight into what is expected with pubertal maturation and what might indicate poor sleep health. A deeper understanding of how sleep health dimensions change during puberty, along with the influencing factors, can lead to better-tailored interventions during this sensitive period.

Biopsychosocial frameworks have been applied to sleep in childhood and adolescence (e.g., Liu et al., 2022; Meltzer et al., 2021). The literature underlines how sleep health during adolescence is influenced not only by individual characteristics but also by contextual and cultural/environmental factors, including social engagement, cultural settings, socioeconomic status, school demands and organization (Bacaro et al., 2023; De Lise et al., 2023; Gariepy et al., 2020; Yeo et al., 2024). However, to our knowledge, this effort has not been applied specifically to puberty, for which changes in sleep have been primarily addressed as consequences of hormonal changes. Understanding the different factors affecting sleep health during puberty is crucial to prevent long-lasting negative consequences.

## Pubertal changes in sleep health

Epidemiological data worldwide indicate an overall deterioration of sleep during adolescence: a high proportion of adolescent girls and boys report overall poor sleep quality. (Galan-Lopez et al., 2021) suffer from excessive daytime sleepiness, (Owens et al., 2020) have late sleep timing and high variability in sleep patterns, (S. J. Crowley et al., 2018) do not sleep for the recommended 8–10 h, and Chaput and Janssen (2016); Malheiros et al. (2021); Wheaton and Claussen (2021) and have poor sleep hygiene habits, such as excessive use of electronic devices at bedtime (Brautsch et al., 2023). The onset of these changes in sleep health is often linked with the onset of puberty (Hoyt et al., 2018; Sadeh et al., 2009).

### Satisfaction

There is data indicating an association between pubertal development and lower subjective sleep quality (Kirshenbaum et al., 2023; Pesonen et al., 2014), while other reports indicate opposite results (Holm et al., 2009). It appears that dissatisfaction with sleep is associated with increased pubertal stage independent of age (Kirshenbaum et al., 2023; Lustig et al., 2021) but more studies are needed to disentagle the interplay between sleep satisfaction, age, and pubertal development.

### Alertness during the day

Daytime sleepiness is common in children and adolescents, with estimated prevalence rates between 10% and 47% (Owens et al., 2020). The prevalence of excessive daytime sleepiness (EDS) increases after puberty (Y. Liu et al., 2019). EDS affects daytime functioning at different levels, influencing executive functions (attention, memory, learning) and mood (Bruni, 2023).

### Timing

The hormonal and physiological changes of pubertal development are associated with a later sleep timing that clashes with early school start time, reducing the sleep window, i.e. the time available for sleep (Carskadon, 2011). Pubertal changes in sleep timing have been observed in different mammalian species and may be driven by reproductive hormones prompting changes in the circadian and homeostatic systems (Hagenauer & Lee, 2012). These systems are the basic processes regulating sleep-wake states: the homeostatic drive represents sleep pressure that increases constantly during wakefulness and dissipates during sleep; the circadian system regulates the timing of different physiological processes, including sleep and wake propension (Borbély, 1982; Borbély et al., 2016). Pubertal maturation is linked to a slower buildup of sleep pressure during waking hours, combined with a delay of melatonin onset (a marker of the circadian rhythm), resulting in a predisposition to stay awake longer and later in the evening (Carskadon, 2011; S. J. Crowley et al., 2018). These changes reflect in a higher prevalence, during adolescents, of an evening circadian typology (e.g., Fischer et al., 2017). The circadian preference, also called chronotype, refers to the individual preferences for sleep-wake timing, i.e. bedtime and waketime, and is a proxy of the endogenous circadian rhythm (e.g., Adan et al., 2012). This preference expresses, at the population level, a continuum that follows a normal distribution: most people are intermediate, while a small percentage is either a morning type or an evening type (Adan et al., 2012, Natale & Cicogna, 2002). This distribution can slightly change with age, with higher prevalence of morning types among children and evening types among adolescents (Fischer et al., 2017). The changes in sleep timing in humans are dramatic: considering a sleep need of 8 to 10 h during adolescence (Hirshkowitz et al., 2015), the delay of about 1 to 2 h observed with puberty onset poses a crucial risk for insufficient sleep (Gradisar et al., 2011). A longitudinal study from childhood to adolescence found that the changes in sleep duration and timing precede the physical changes associated with puberty (Sadeh, R. Dahl et al., 2009). Moreover, the differences between sleep timing during weekdays and weekends, or *social jetlag* (Wittmann et al., 2006), shows a dramatic increase with age from puberty to adolescence (Laberge et al., 2001). The delay and lengthening of the sleep period on weekends are associated with alterations in natural sleep-wake regulation, observable as sleep onset and maintenance difficulties (Wittmann et al., 2006).

### Efficiency

Sleep efficiency deteriorates during adolescence due to poor sleep habits (e.g., Zambotti et al., 2017). However, it is not clear if sleep efficiency is associated with pubertal development. One study did not find a significant association between pubertal status and actigraphy-derived sleep efficiency (Kirshenbaum et al., 2023). Overall, the literature underlines a discrepancy between subjective sleep satisfaction and physiologically assessed sleep efficiency in adolescents (Kirshenbaum et al., 2023; Short et al., 2012).

### Duration

Given the delay in sleep timing, it is not surprising that sleep duration is often reduced during pubertal development. The National Sleep Foundation recommends adolescents to get 8–10 h of sleep per night (Hirshkowitz et al., 2015). This standard is rarely met by adolescents (e.g., Wheaton & Claussen, 2021). However, literature has found contradicting results, with some reports finding no significant association between pubertal development and actigraphy-derived sleep duration (Kirshenbaum et al., 2023; Pesonen et al., 2014). For instance, Kirshenbaum et al. (2023) found a null association between sleep duration and pubertal stage in a USA sample of boys and girls. Authors suggested that this finding could have been influenced by a low rate of insufficient sleep in the sample, especially considering that the school-start was not earlier than 8:30 am (Kirshenbaum et al., 2023).

### Sleep-related behaviours

Regarding sleep-related behaviours, most data focus primarily on sleep pattern consistency and sleep hygiene practices, while bedtime routines have been less investigated in puberty and adolescents compared to childhood. Sleep hygiene refers to different behaviours that can positively or negatively influence sleep, i.e. facilitate or inhibit good quality sleep (e.g., Jansson-Fröjmark et al., 2019). Poor sleep hygiene includes using technology before bed, sleeping in a room that is too hot/cold or noisy, and engaging in pre-sleep activities that increase anxiety or stress (De Pasquale et al., 2024). Sleep-related behaviours are closely linked to the healthy status of the other sleep health dimensions (Meltzer et al., 2021). For instance, Galland and colleagues (2017) found that in adolescents a 1-hour increase in evening technology use time was associated with a 20% increased odds of poor sleep efficiency (Galland et al., 2017). For children, the role of consistent bedtime routine in promoting sleep health is well-established (Mindell & Williamson, 2018). More longitudinal data are needed tracking changes in bedtime routines from childhood to adolescence to highlight their role in mitigating the risk of poor sleep health during pubertal development.

Overall, the literature points to pubertal-related physiological changes in sleep-wake regulation (see Table 1) that can increase the risk of sleep difficulties and unhealthy sleep, in conjunction with sleep habits and social demands.

**Table 1.** Main changes in sleep health dimensions associated with pubertal development

Sleep health dimensions	Definition	Pubertal changes
Sleep satisfaction/quality	The subjective assessment of one’s own sleep quality/The multidimensional assessment of the quality of sleep	Poor subjective sleep quality, with a discrepancy between self-reported sleep satisfaction and physiological measures of sleep quality
Daytime alertness (vs sleepiness)	The ability to sustain attentive wakefulness during the day, as opposed to daytime sleepiness	Excessive daytime sleepiness is common in children (10%-47%) and adolescents and increases after puberty
Sleep timing	The allocation of the main sleep episode in the 24-hour day	Delay in sleep timing due to hormonal changes affecting the circadian rhythm and sleep pressure. Behavioural and social factors contribute to late sleep timing.
Sleep efficiency (or continuity)	The ability to fall asleep easily, to return to sleep after a night-time wake and to remain asleep until the desired waketime	Sleep efficiency declines after puberty; however, evidence do not clearly show a link between puberty and actigraphy-derived sleep efficiency
Sleep duration	The amount of sleep in the 24-hour day	Decrease in sleep duration due to later sleep timing and early school schedule.
Sleep-related behaviours	Pre-sleep activities compatible with sleep, consistent bedtime routines, and regularity of sleep habits	Sleep pattern variability (weekdays vs weekends) increases and sleep hygiene deteriorate with age from puberty to adolescence. Sleep hygiene and bedtime routines are under investigated in association with puberty.

## A perspective on multilevel factors

The most notable changes in sleep health during adolescence are the delay in circadian rhythm, with increased preference for later sleep and wake hours (i.e., eveningness), and the decrease in sleep pressure, leading to later sleep timing and sleep insufficiency (e.g., S. J. Crowley et al., 2018). The changes in sleep health, prompted by pubertal development, are a result of complex interactions between hormonal changes, environmental factors (social media use, school schedule, family factors) and psychological aspects, including the emergence of increased independence and new social roles (S. J. Crowley et al., 2018; Ricketts et al., 2022; Tarokh et al., 2016). Overall, the deterioration of sleep health with the passage to adolescence has multifactorial causes, both internal and external (Owens & Weiss, 2017). Biopsychosocial and contextual models have been applied to children (Covington et al., 2021; El-Sheikh et al., 2017) and adolescents (Becker et al., 2015; S. J. Crowley et al., 2018) sleep, assessing the different biological (e.g., genetic influence, hormonal changes), psychological (e.g., mental health, emotional problems), social and contextual factors (e.g., familiar factors, peer relationships, social media use) influencing sleep. These approaches increase the understanding of the complexity of sleep trajectories during development, following the rapid changes and shifts at different levels.

**Figure 1.**
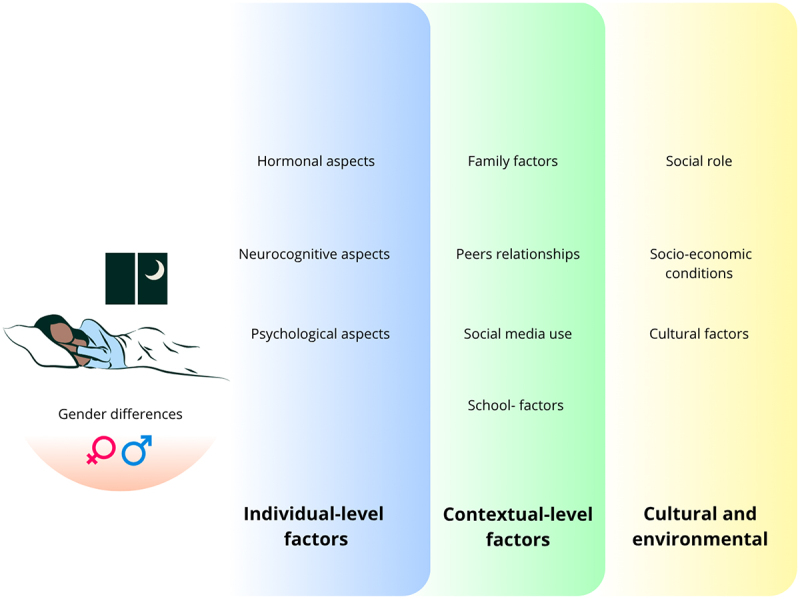
Multilevel factors affecting sleep health during pubertal development.

The factors associated with sleep health in puberty are here declined by individual-level (hormonal, neurocognitive, and psychological), contextual-level (peers, family, and school), and cultural/environmental-level (social role, socioeconomic conditions and cultural aspects). Moreover, sex differences can shape the impact of the different factors and are thus treated separately. Figure 1 offers an overview of these factors. While these multilevel factors are reported separately, they are considered from a systemic perspective and thus posited to interact reciprocally. In addition to examining the effects of these factors on sleep health, we acknowledge the literature’s indication of bi-directional associations. We will highlight this in certain instances, as these mutual influences have a great impact on sleep health.

### Individual-level factors

#### Hormonal aspects

Pubertal hormonal changes involve sex hormones (testosterone and oestrogen), but also melatonin and growth hormone, which contribute to the regulation of physiological processes such as sleep (Colrain & Baker, 2011; Lucien et al., 2021). Sex steroids, both testosterone and oestradiol, can influence sleep patterns (Dorsey et al., 2021; P. Y. Liu, 2019), although data on the effect of irregular testosterone levels are not consistent (P. Y. Liu, 2019). The fluctuations in oestradiol associated with the menstrual cycle can disrupt sleep in girls, especially when accompanied by more painful symptoms (e.g., Pengo et al., 2018). Together with oestrogens, other menstrual hormones, such as luteinizing hormone, follicle-stimulating hormone, and progesterone, can contribute to the increase in sleep disturbances in girls during the week before the onset of menses (Alzueta & Baker, 2023; Haufe & Leeners, 2023). On the other hand, there are data indicating that hormones can also impact boys’ sleep. For instance, the increase in testosterone levels during pubertal maturation in boys has been associated with alterations in sleep architecture (i.e., the structural organization of sleep, including the different stages and cycles), especially in REM sleep (Wittert, 2014). Moreover, increased levels of testosterone during puberty appears to delay sleep timing and affect specifically circadian rhythms but not sleep duration (Wittert, 2014). However, studies examining the effects of testosterone levels on specific dimensions of sleep health among pubertal boys are lacking. Further research is needed to address this issue.

The rapid changes in growth hormone (GH) lead to the physical signs of puberty as a “growth spurt”. This hormone is predominantly secreted during deep sleep, especially in the first slow wave sleep episode after sleep onset (Takahashi et al., 1968). While extensive literature has investigated the detrimental effect of poor sleep health on the secretion of GH, recent evidence also indicates that idiopathic GH deficiency can negatively affect sleep in children, suggesting a complex relationship between sleep and GH (Zaffanello et al., 2024).

#### Neurocognitive aspects

The onset of adolescence is accompanied by neurocognitive changes that are bidirectionally linked to psychological and sleep health (M. E. Mitchell & Nugiel, 2024). The biological changes associated with puberty are also linked to significant structural changes in the brain (Kretzer et al., 2024; Vijayakumar et al., 2018). At this stage, aspects of brain development are unique compared to adolescence. For instance, at the expected start of puberty there is a peak in the volume of grey matter in the frontal and parietal lobes, which shows a decrease later in adolescence (e.g., Giedd et al., 1999). The influence of pubertal changes on the developing brain can be one mechanism explaining the heightened vulnerability to mental health problems (Vijayakumar et al., 2018). Indeed, individual differences in these developmental changes have been longitudinally associated with the severity of mental health problems (Cropley et al., 2021). Neurocognitive changes also directly influence sleep-wake regulation. Synaptic pruning, i.e. the physiological decline in cortical synaptic density from late childhood to early adulthood, is accompanied by a decline in the amplitude of slow wave activity (SWA) (de Vivo et al., 2014; Kurth et al., 2010). The rate of decline in delta power during sleep has been linked to pubertal development (Campbell & Feinberg, 2009; Campbell et al., 2012) and can contribute to daytime sleepiness in adolescents (Campbell et al., 2007).

#### Psychological aspects

The transition from childhood to adolescence involves significant changes at neurobiological, hormonal, social, and contextual factors, which are relevant for both sleep health and emotional functioning (Anastasiades et al., 2022; Diao et al., 2020; Lucien et al., 2021). Adolescence is characterized by an increase in emotional and behavioural problems (Solmi et al., 2022). This vulnerability is particularly pronounced in cases of early puberty compared to peers (Sequeira et al., 2017). Early puberty can lead to heightened stress due to physical and psychological changes and peer comparison (Diao et al., 2020; Mastorci et al., 2024), exacerbating difficulties in social adaptation and psychological well-being (Diao et al., 2020).

Puberty has been specifically linked to increased vulnerability to anxiety and depression, especially in girls (Hyde & Mezulis, 2020; Karl et al., 2024; M. E. Mitchell & Nugiel, 2024). Various mechanisms explain this heightened vulnerability. Increased levels of sex hormones (oestrogen and testosterone) are associated with mood reactivity and heightened sensitivity to stress during pubertal development (Lucien et al., 2021). Additionally, during adolescence, there is an increased sensitivity to social and emotional cues (e.g., Ferri et al., 2014). Particularly in girls, pubertal development introduces new challenges in the social context, impacting self-image and increasing exposure to stressors (Pfeifer & Allen, 2021). Interpersonal stress, physical changes, and body-image dissatisfaction can increase the risk of psychopathological problems during puberty (Jiang et al., 2021; McGuire et al., 2019). These stressors impact a cognitive system that is still maturing, particularly in areas involved in emotional regulatory processes (i.e., the functional connectivity between limbic structures and the prefrontal cortex) (Karl et al., 2024; Steinberg, 2008). Emotion regulation, i.e. the ability to change the emotional experience to pursue personal goals or to adapt to the context (Gross, 1998), is a key contributor to overall mental health (Aldao et al., 2016; Alloy et al., 2016).

The psychological changes happening with puberty can impact sleep. Indeed, psychological well-being and emotional processes in youths are closely linked with sleep health (Bacaro et al., 2024). For example, up to 90% of anxious youth experience impairments in sleep health such as sleep dissatisfaction, daytime sleepiness, reduced sleep efficiency, and insufficient sleep duration (McMakin & Alfano, 2015). One putative mechanisms linking sleep to psychological health is emotion regulation (e.g., Meneo et al., 2023). Lustig et al. (2021) found that among adolescents aged 8–15 years, sleep dissatisfaction was associated with difficulties in emotion regulation, with pubertal development in girls serving as a significant moderator of this relationship (Lustig et al., 2021). Data suggest that depressive and anxiety symptoms can contribute to daytime impairments in adolescents, including daytime sleepiness, fatigue, and attention deficits (Cavalcanti et al., 2021; Y. Liu et al., 2019). Sleep continuity can be disrupted by the puberty-related increase in rumination and worry over daily experiences and problems (Alloy et al., 2016; Li et al., 2024; Rapee et al., 2019). In turn, this processes are strictly associated with anxiety and depression (Olatunji et al., 2013; Visted et al., 2018). The effects of psychological status on sleep health dimensions can contribute to insufficient sleep duration in youth with anxiety and depression (Haugland et al., 2021; J. Zhang et al., 2017). Furthermore, longitudinal data indicate that internalizing and externalizing problems during adolescence can predict short sleep duration, suggesting a causal role of psychological well-being on this dimension of sleep health (Bacaro et al., 2024).

### Contextual-level factors

#### Family-level factors

The role of the family context in shaping children’s sleep habits has been well-established (Bacaro et al., 2019; Sadeh & Anders, 1993; Sadeh, Mindell, et al., 2009). Parents’ involvement in children’s routines, their perception and expectations, and their own sleep habits influence sleep during childhood (e.g., Bacaro et al., 2019). Moreover, other factors, such as parent-infant bonding and parents’ psychological well-being, can also influence children’s sleep from early development (Sadeh & Anders, 1993). With puberty, family dynamics often shift, with some factors continuing to exert an influence beyond childhood, and others emerging at puberty. A meta-analysis identified the main family-level factors contributing to adolescents’ sleep health: parental rule-setting for bedtime and parent sleep behaviours, healthy parental sleep and family functioning and parental warmth (Khor et al., 2021). Recent studies also show that adolescents sleep longer and better when perceiving positive family interactions (S. Bai et al., 2022), and that their sleep patterns closely correlate with those of other family members (Reynaud et al., 2024).

There is evidence that family functioning from puberty to adolescence can have long-term effects on sleep health (Kelly et al., 2024; Leonard & Khurana, 2022). For instance, parental involvement can reduce media use at bedtime, increasing sleep duration (Leonard & Khurana, 2022). Moreover, exposure to family conflicts in the form of frequent and intense conflicts between parents or between parents and the adolescent can increase stress or directly disturb sleep, increasing adolescents’ sleep difficulties (Leonard & Khurana, 2022) and potentially affecting emerging adults’ sleep efficiency in the long term (Kelly et al., 2024). On the other hand, the emotional climate in the family has been shown to longitudinally mediate the impact of low socioeconomic status on children’s and adolescents’ sleep (Doane et al., 2019; Philbrook et al., 2020).

Family structure is another possible factor influencing sleep in the developmental age, although this has been less studied in puberty. For instance, a cross-sectional study of a large sample of adolescents (*n* = 8833, 16–18 years old) found that those living in single- and stepparent families had a higher risk of insufficient sleep, delayed-sleep phase disorder and insomnia symptoms compared to peers in nuclear families (Nilsen et al., 2022).

Overall, familiar influence on sleep health seems to extend beyond childhood into adolescence. In this transition, the impact of shifts in family dynamics during pubertal development can represent a critical point for the trajectories of adolescents’ sleep health that merit further investigation.

Moreover, changes in sleep patterns during puberty can also influence family dynamics. A longitudinal study tracking pubertal development and changes in sleep patterns showed that pubertal status and tempo predicted short sleep duration and variability in total sleep time in adolescents, which in turn increased family conflicts (Peltz et al., 2024). Family conflicts were also found to interact with pubertal development at 12 years old in predicting eveningness one year later in girls, but not in boys (Díaz Morales et al., 2023). These findings suggest that familiar factors are bidirectionally linked with sleep changes during puberty.

#### Peer-level factors

In the transition from childhood to adolescence, there is a shift toward higher value placed on peer relationships and increased social engagement (Laursen & Veenstra, 2021). For this reason, peer stress evaluation acquires a major role in emotional well-being of boys and girls (Oberle et al., 2025; Sandre et al., 2023). Thematic analyses also show the relevance of peer stress and fear of missing out for adolescents’ sleep, as boys and girls reports these factors as main barriers to adequate sleep (Yeo et al., 2024). Biopsychosocial changes associated with pubertal maturation increase the sensitivity to exclusion, conflicts, and absence of social support (e.g., Laursen & Veenstra, 2021). The effect of peer stress on mental well-being appears particularly evident in girls, who tend to have a stronger depressive response to peer stress than boys (Conley & Rudolph, 2009; Giulietti et al., 2022). Being puberty a potentially stressful experience, especially for those with early puberty, the level of peer stress might exacerbate the adverse effect of puberty on mental health (Benjet & Hernández-Guzmán, 2002; Sontag et al., 2008; Vijayakumar et al., 2024). Accordingly, feeling accepted vs isolated by peers outside and inside the school context is associated with sleep quality in adolescents (Benzi et al., 2023; Fabris et al., 2024; Maume, 2013). A longitudinal study from childhood to pre-adolescence (8–11 years old) showed that high loneliness in mid-childhood is associated with sleep problems during pre-adolescence even when loneliness level reduces (Harris et al., 2013), highlighting the need for early detection of vulnerable teens.

On the extreme of peer stress, peer victimization is associated with increased mental health and sleep problems, including low sleep efficiency (i.e., problems falling or staying asleep), among children and adolescents (van Geel et al., 2016). Bullying behaviours become a key risk factor for adjustment after pubertal development. Being the victim of bullying behaviours, including verbal, physical, social, and cyberbullying, increases the likelihood of experiencing overall low sleep quality, difficulties in falling or staying asleep (sleep efficiency), and short sleep duration (Ding et al., 2023; Donoghue & Meltzer, 2018; Hysing et al., 2019). Different pathways link victimization and sleep health, such as fear of future victimization, rumination, and environmental stress, which increase bedtime arousal (Donoghue & Meltzer, 2018). In turn, disruption in sleep continuity can lead to emotion dysregulation, heightening anxiety, depression, and negative evaluations of others (Donoghue & Meltzer, 2018). Although much research on peer victimization has been conducted across all stages of adolescence, studies on early adolescents suggest that puberty may be a more sensitive period for the negative impact of bullying on sleep and mental health (Ding et al., 2023; Tu et al., 2019).

In addition to peer stress, the heightened value places on peer relationship can also influence beliefs and attitudes about sleep. A qualitative analysis emphasized that late bedtimes are not perceived as a problem by adolescents, but rather as a personal choice (Kosticova et al., 2024). A focus group study also showed the role of perceived norms in adolescents’ sleep, finding that most adolescents assumed their peers had insufficient sleep duration and poor sleep quality, with long sleep onset times (Vandendriessche et al., 2022). Peer influence on attitudes toward sleep merits further consideration, as peers become primary source of information during adolescents, with potentially great impact on knowledge and beliefs regarding sleep.

#### Social media use

The use of electronic devices near bedtime and in bed has been a focus of research on adolescent sleep functioning for decades. Watching television, using smartphone to chat, videogames, and other activities involving electronic devices are associated with impaired sleep health, including increased daytime sleepiness, difficulties in falling asleep, delayed sleep timing, and shortened sleep duration (for an overview, see Brautsch et al., 2023; Lund et al., 2021; Pagano et al., 2023). Research suggests that the association between electronic media use and sleep health may be bidirectional: using electronic devices at night lead to unhealthy sleep pattern (delay in sleep timing, disrupted sleep continuity, reduced sleep duration); on the other hand, adolescents having difficulties to fall asleep may use electronic devices as a mean to cope or to aid sleep (Daniels et al., 2023; Eggermont & Van Den Bulck, 2006). Both directions appear supported by the literature, although the impact of media use on sleep has received more support from longitudinal studies. The use of electronic devices at night in early adolescence is associated with poorer sleep health in mid- and late-adolescence (Pagano et al., 2023). The impact of media use at bedtime on sleep is imputed to different mechanisms: the direct displacement of sleep; the effects of blue light at night on circadian regulation; the increase in psychological arousal (Cain & Gradisar, 2010; Pagano et al., 2023).

A particular use of electronic devices is for social media contents. Social media use has become one of the most prevalent leisure activity for adolescents, extending throughout the day (Kuss & Griffiths, 2017). The frequency of social media use during the day and especially close to bedtime can impact on sleep health through several mechanisms, coupled with the negative effect of electronic devices use (Carter et al., 2016; Pagano et al., 2023; Scott et al., 2019). Social media content and activities (i.e., chatting, viewing posts) can increase emotional and mental arousal, either positive or negative, and this effect also renders them highly engaging (Schreiner et al., 2021). Moreover, the biopsychosocial changes associated with puberty can make this period more vulnerable to negative feedback from social media use, such as body comparison (Petro et al., 2025). Additionally, as a high proportion of adolescents keeps their phone turned on at night, sleep may be interrupted by notifications (Brautsch et al., 2023). Some adolescents also report difficulties in disengaging from social media before bed due to fear of violating social expectations (Scott et al., 2019). The rise in social media use at night in younger teens is emerging problems that needs to be addressed to promote sleep health during the transition from childhood to adolescence (Hale et al., 2019).

To understand motivations and processes maintaining social media use at night, this behaviour should be put into the broader social context of adolescents. The use of social media at night can be a mean for socialization and be associated with fear of missing out on occasions of social interactions (Charmaraman et al., 2021; C. Gupta et al., 2022; Hilty et al., 2023). The need to feel included and to belong to a safe relational context is a basic drive behind adolescents’ behaviours, and when frustrated, it leads to feelings of isolation, rejection, and emotional difficulties (Arslan, 2021). Thus, adolescents may compensate by increasing their contact in the online world, i.e., through social media (Valkenburg et al., 2005). A sense of belonging on social media but not within their peer group, is indeed associated with a higher risk of social media addiction, school maladjustment and psychological difficulties (Fabris et al., 2024). In turn, excessive or problematic social media use and social media addiction are associated with poor sleep health including displacement of sleep, sleep insufficiency, anxiety about being available, and increased arousal at night (Alonzo et al., 2021; Cain & Gradisar, 2010; Vahedi & Saiphoo, 2018).

Parental influence can mediate the impact of media use at night on sleep. For instance, van den Eijnden et al. (2021) and colleagues found that parental rules about media use at bedtime may prevent the longitudinal negative impact of social media use on sleep health. However, this protective effect was not significant for teens who were already highly engaged social media users (van den Eijnden et al., 2021).

Studies on adolescents highlight the importance of considering the influence of peer relationships and parental rules in adolescents’ use of social media at night. However, there is a need for more longitudinal studies specifically investigating trajectories of sleep, social media use and its social determinants in the transition from childhood to adolescents, i.e., during puberty.

#### School-level factors

Among the school-level factors influencing sleep health, the most studied are school start time and school demands. Pubertal shifts in circadian rhythms and social factors lead to a later bedtime that clashes with early school start time (Carskadon, 2011; S. J. Crowley et al., 2018). This mismatch reduce time available for sleep, increasing odds of sleep deprivation and excessive daytime sleepiness, and contribute to circadian misalignment between teens internal clock and the social clock (Carskadon, 2011; S. J. Crowley et al., 2018; Wheaton et al., 2016). A recent comparison between two school schedules of teens aged 11–18 years showed that bedtime was not different between students while those under earlier school start time had shorted sleep duration and increased mood disturbance during the day (Singh et al., 2023). This evidence inform recommendation to reconsider a later school-start time for adolescents (e.g., Ramar et al., 2021), although this do not exclude an approach aimed at improving sleep timing and sleep habits.

With the start of adolescence, boys and girls face an increase in academic demands. School demands can increase stress and trigger excessive worry and rumination, negatively effecting sleep initiation and maintenance (i.e., efficiency) and mental well-being (Deng et al., 2023; Vandendriessche et al., 2022; W. Zhang et al., 2020). School stress is often mentioned among the barriers to healthy sleep in qualitative analyses of adolescents’ sleep (Yeo et al., 2024) and is associated with reduced perceived sleep quality (Thapa et al., 2025).

Overall, school-start time and school demand have been investigated mostly in association with sleep duration, daytime sleepiness and impairment in sleep efficiency. Lesser is known on how school factors influence sleep timing in adolescents, apart from the known effect of earlier rise time. Moreover, how school-factors influence the perceived satisfaction about sleep is also an area that should be more investigated.

### Cultural and environmental

#### Social role

The term social role is here used to refer broadly to the set of expected behaviors, responsibility, and norms associated with a particular position in society (e.g., Thoits, 1983). With puberty, there is a significant shift in social role(s): when children transition into adolescence, their role in the family system, in the peer system, and in the broader society changes (Albarello et al., 2018). Most research on the social aspects of sleep health during adolescence have investigated the pivotal role of peer relationship and changes in family dynamics (De Lise et al., 2023), while less is known about the influence of the broader society.

The visible morphological changes associated with puberty can alter how others respond, not only in the family and peer context but also in the larger context, increasing self-awareness and impacting self-concept (Rapee et al., 2019). For instance, with the development of secondary sex characteristics, girls are more subjected to sexualisation, while boys are viewed as more independent and less in need of support than before (Mendle, 2014; Mendle & Ferrero, 2012). The increased social expectations can contribute to emotional problems and confusion in self-concept (Rapee et al., 2019), which can, conversely, negatively affect sleep health.

However, research on how the change in social role affect sleep health specifically are lacking. Social norms include what a person perceived to be expected as a typical and appropriate behavior, with importance influence on how that person actually behave (Mackie et al., 2015). This could potentially have great influence on sleep behaviors of pubertal boys and girls. Among the social role factors, the expectations about independence, norms on lifestyle factors, and belief-systems acquired from the larger social context can all be hypothesised to influence sleep health. How this changes affect sleep health needs to be addressed.

#### Environmental factors: socio-economic conditions and cultural aspects

Environmental aspects associated with sleep health in young populations include socio-economic status of parents and cultural practices around bedtime, including sleeping arrangement (e.g., Jeon et al., 2021). Other factors include those already discussed in this review, such as school start time and family organization.

Regarding socio-economic status (SES), children and adolescents from low SES contexts are more likely to exhibit unhealthy sleep habits, insufficient sleep, poor sleep quality, low sleep efficiency, and greater daytime sleepiness (Felden et al., 2015; Hysing et al., 2017; Philbrook et al., 2020). A recent meta-analysis on 46 studies on children showed that different aspects of parental socioeconomic status were significantly associated with children’s sleep health: higher parental education was associated with longer sleep duration and better sleep quality (Cameron et al., 2022). Household income showed significant association with sleep quality but not with sleep duration, except in studies with higher quality and proportion of White children in the sample (Cameron et al., 2022). Another systematic review assessed the impact of socioeconomic factors on sleep health through the lifespan, showing that parental socioeconomic indicators (income, family SES, educational level) are associated with children’ and adolescents’ sleep quality, daytime alertness, efficiency (i.e., sleep initiation and maintenance problems), and duration (Etindele Sosso et al., 2022). Studies also indicate that sleep is more vulnerable to the effect of neighbourhood disadvantage in the paediatric population compared to adults (Billings et al., 2020). For instance, children from low SES neighbourhood report shorter sleep duration and higher variability in sleep timing compared to peers living in more advantaged neighbourhoods (Billings et al., 2020). Low SES neighbourhoods are often marked by greater air pollution, lower social cohesion and perceived safety, less proximity to green spaces, and increased density of population, all affecting sleep opportunity and quality in children and adolescents (Billings et al., 2020). Socioeconomic disparities in youth’s sleep can be imputed also to other mechanisms, including lower control over environmental conditions e.g., space), higher sensory stimulation (e.g., noise), lack of structured bedtime routines, perceived neighbourhood safety, and pressure from economic hardships in the family (Philbrook et al. 2020; Zeringue et al. 2023). Additionally, different ecological sites (urban vs rural) have different levels of light exposure at night, which can also affect sleep efficiency of adolescents (Silva-Caballero et al., 2023).

Cultural factors have an important influence in shaping practices around bedtime in young population, especially in children, while less has been investigated in early adolescents and in relation to pubertal development (Jeon et al., 2021). For instance, a systematic review on 25 comparative studies in children and adolescents showed that those from Europe, North America and Australasia tended to report earlier bedtime, earlier wake up time and longer sleep duration than youths from Asia and the Middle East region (Jeon et al., 2021). Silva-Caballero et al. (2023) also found a differential impact of co-sleeping vs solitary sleeping in early adolescents based on cultural norms: while co-sleeping is not common in adolescents, when it is a cultural practice it is associated with better sleep efficiency; on the other hand, solitary sleeping in the urban environment, which is a more common practice, was also associated with better sleep efficiency.

Taken together, this evidence underly the need to consider different aspects of the environment together with social norms when considering sleep behaviors and sleep health in adolescents.

## Gender differences in sleep health during puberty

Gender differences in sleep have been mostly investigated in insomnia research, as the prevalence of sleep problems is higher in women as compared to men across the lifespan (Pengo et al., 2018). Pubertal maturation is considered a precipitating factor for sleep difficulties in girls, due to hormonal changes, psychological adjustment and social factors (for a comprehensive review, see Franco et al., 2020). While some sex differences in sleep exist prior to puberty, they become more pronounced during puberty (Franco et al., 2020; Roenneberg et al., 2004), specifically with the first menstrual cycle, i.e. menarche (Pengo et al., 2018). The significant physical and psychological changes associated with puberty mark the onset of most sex-related differences in sleep health (Meers et al., 2019).

### Sleep satisfaction

Although research on sex differences in sleep satisfaction during puberty is limited, evidence suggests that females tend to report greater dissatisfaction with their sleep (Knutson, 2005; Lustig et al., 2021; Matos et al., 2019). Pubertal development in adolescent girls is linked to a higher risk of frequently waking up tired, a trend that increases with age (Knutson, 2005). In a study on 3869 adolescents (13–19 years old), girls report waking up tired more often than boys (Matos et al., 2019). These findings are supported by a recent study on 256 children and adolescents aged 8 to 15, which found that pubertal development is associated with greater sleep dissatisfaction in girls (Lustig et al., 2021).

### Alertness during the day

Girls are more likely than boys to report experience daytime sleepiness, including falling asleep more easily in the afternoon, an index of increased sleep pressure (Matthews et al., 2014; Munezawa et al., 2011). For example, a large study by Gaina et al. (2007) involving 9261 preadolescents aged 12–13 years found that girls experienced more daytime sleepiness than boys (Gaina et al., 2007). More recently, a cross-sectional study on 10,086 children and adolescents aged 6 to 18 years confirmed a female predominance in excessive daytime sleepiness after mid-puberty (Y. Liu et al., 2019).

### Timing

The literature points to sex differences in preadolescents in sleep timing, although the direction of the difference is not clear (Meneo et al., 2025). Some studies have found that girls sleep longer on weekdays (Russo et al., 2007) and weekends (Collado Mateo et al., 2012; Natal et al., 2009; Russo et al., 2007; Yang et al., 2005), while others report no such sex differences (Borchers & Randler, 2012; Lee et al., 1999). One actigraphic study indicated no sex differences in bedtime (Gaina et al., 2005). Instead, wake-up time showed a significant difference, with girls waking up earlier than boys (Gaina et al., 2005). Another study found later rising time on weekends and earlier rising time on weekdays in girls compared to boys (Collado Mateo et al., 2012). Moreover, girls tend to have more irregular sleep patterns, with longer time in bed (TIB), longer sleep duration and later wake-up times during weekends, and greater weekday—weekend TIB discrepancies compared to boys (Laberge et al., 2001; L. N. Lin et al., 2017).

These differences appear to be related to females’ higher pubertal stages when compared to boys of the same chronological age (Matos et al., 2019). Since girls typically start puberty at an earlier age than boys, they also begin to show circadian phase advancement and a delay in sleep timing before boys (Hagenauer et al., 2009; Laberge et al., 2001; Petersen et al., 1988; Roenneberg et al., 2004; Tonetti et al., 2008). Overall, while findings on sex differences in pubertal sleep timing are mixed, evidence suggests that girls tend to exhibit greater variability in sleep patterns compared to boys, i.e. longer weekend sleep duration, a greater difference in sleep schedules during weekdays and weekends, and earlier pubertal-related shifts in sleep timing.

### Efficiency

Sleep continuity is also affected by pubertal development: while preadolescent girls have higher sleep efficiency than boys (Gaina et al., 2005), this trend reverses with the onset of puberty (Smidt et al., 2021). Indeed, female adolescents tend to report more nighttime awakenings, lower sleep efficiency (Lee et al., 1999; Matthews et al., 2014; Vollmer et al., 2017), and more trouble falling asleep and/or maintaining sleep (Kocevska et al., 2020; Vollmer et al., 2017) compared to male adolescents.

### Duration

Research indicates that pubertal development is negatively associated with sleep duration for both sexes (Knutson, 2005). However, puberty-related sex differences are not clear. Some studies report no gender differences in self-reported sleep duration (Kocevska et al., 2020; Lytle et al., 2011; Williams et al., 2013), while others indicate longer sleep duration among girls, both self-reported (Franco et al., 2020; Pollak & Bright, 2003) and assessed with actigraphy Matthews et al. (2014); J. A. Mitchell et al. (2020); Short et al. (2012). However, several other studies have consistently observed shorter self-reported sleep duration among girls (Bauducco et al., 2016; Keyes et al., 2015; Lewin et al., 2017; L. N. Lin et al., 2017; Munezawa et al., 2011; Seo et al., 2017; Vollmer et al., 2017). According to Franco et al. (2020), pubertal development can lead to increased weekends sleep duration in girls, indicating a possible increased need to catch up on weekdays lost sleep as compared to boys.

### Sleep-related behaviours

Research exploring gender differences in sleep-related behaviours in preadolescents is lacking. At least one study (R. Gupta et al., 2016) found no significant sex differences in the most common pre-sleep activities among 8-to-13-year-olds, which included reading books, watching TV, and playing games on mobile phones. However, more girls tended to follow their siblings to bed and often required a specific object to help them fall asleep (R. Gupta et al., 2016). Additional studies are needed to deepen our knowledge about sex differences in pre-sleep activities during puberty and adolescence.

In summary, the literature highlights gender differences in some dimensions of sleep health during puberty, with female adolescents appearing to be more likely to experience impaired sleep health than their male counterparts (Smidt et al., 2021). Females tend to report lower sleep satisfaction (Knutson, 2005; Lustig et al., 2021; Matos et al., 2019) and higher levels of daytime sleepiness (Gaina et al., 2007; Matthews et al., 2014) compared to their male peers. Moreover, girls experience reduced sleep efficiency, with increased nighttime awakenings (Smidt et al., 2021). While findings on sleep timing are mixed, girls generally exhibit greater variability in sleep patterns (Laberge et al., 2001; L. N. Lin et al., 2017). Results of studies on sleep duration during puberty are mixed, as well, with several studies reporting shorter sleep among girls (Bauducco et al., 2016; Keyes et al., 2015) and others finding no sex differences (Kocevska et al., 2020; Lytle et al., 2011). In the end, certain areas, such as gender differences in pre-sleep behaviours, remain underexplored and warrant further investigation to fully understand the gender-specific trajectories of sleep health during this critical developmental period.

Most results are derived from studies involving samples of a wide age range, and this makes it challenging to highlight the effect of pubertal status on sleep health. Further research focusing specifically on pubertal samples is needed. Puberty appears to be a critical stage for the emergence of sex differences in sleep health that could have lifespan implications, given that sleep problems are more common in adult women than men (Mong & Cusmano, 2016).

### Factors affecting sleep health in pubertal girls

During puberty, many factors may be involved in sex-related sleep differences, from hormonal to psychological and social (Baglioni & Palagini, 2022; Franco et al., 2020; Mong & Cusmano, 2016).

#### Biological factors

One factor contributing to observed sex differences in sleep timing and duration is the different rates of pubertal maturation between girls and boys.

First, the phase delay in circadian rhythm occurs earlier and more rapidly in girls than boys (e.g., Hagenauer et al., 2009).

Second, puberty-related sex differences in stress hormones might also be a contributing factor to observed differences in sleep satisfaction and complaints (Franco et al., 2020). Previous research indicates that sex differences in the activity of the hypothalamic-pituitary-adrenal (HPA) axis, which plays a key role in stress response, become more pronounced with pubertal maturation (Stroud et al., 2011). The HPA axis activity and sleep are bidirectionally linked: sleep loss increases the HPA axis activity, which in turn may impair sleep (Balbo et al., 2010). The HPA system has been correlated with wakefulness and circadian rhythmicity, suggesting the possibility that HPA axis activity could influence the circadian system regulating sleep (Dorsey et al., 2021). Females typically exhibit a stronger stress response than males (Dorsey et al., 2021). This heightened reactivity may contribute to increased arousal and a stronger wake drive in females (Dorsey et al., 2021), potentially explaining part of the sex difference in sleep patterns. Additionally, disrupted sleep is linked to elevated HPA axis activity upon waking in adolescents (J. Zhang et al., 2014), suggesting that changes in HPA axis activity across puberty may underlie the emergence of sex-specific vulnerabilities to poor sleep quality (Franco et al., 2020). Finally, the fluctuation of ovarian hormones across the menstrual cycle further contributes to a higher risk of poor sleep health in adolescent girls (Campbell et al., 2012). However, more research is needed to fully understand how the neuroendocrine system interacts with sleep-wake regulatory circuits to increase the risk of poor sleep health in women (Mong & Cusmano, 2016) and particularly in adolescent girls.

#### Psychological factors

Puberty is associated with an increased risk of mood disorders and emotional problems in girls (Mendle et al., 2020; Viner, 2015). Emotional problems, in turn, are strictly tied to sleep health (Meers et al., 2019). Puberty can be a stressful period for girls, who are more likely than boys to experience body dissatisfaction and peer-related stressors, with negative consequences on psychological health (Conley & Rudolph, 2009; Ge et al., 2001; Hamlat et al., 2014). The greater tendency to rumination in girls compared to that of boys can contribute to increased risk of depressive symptoms, especially in association with early puberty (Alloy et al., 2016). Moreover, rumination, worry, and intrusive thoughts, especially when occurring at bedtime, could interfere with the process of falling asleep and maintaining sleep, as well as with the perception of sleep duration and quality (Perlis et al., 2001).

Individuals experiencing sleep difficulties also report higher levels of dysfunctional traits, such as perfectionism and neuroticism (Gurtman et al., 2014; Van De Laar et al., 2010). Notably, high perfectionism is prevalent during adolescence and appears to be more prevalent among females (Richardson & Gradisar, 2020). Cognitive arousal (worry and rumination), affective arousal (anxiety and depression), and emotion regulation seem to play a mediating role between perfectionism and sleep difficulties (Schmidt et al., 2018). However, the interaction of personality factors, sleep and sex in puberty has been under investigated.

Also, body and weight dissatisfaction are important factors during this developmental stage (J. Lin et al., 2025; White et al., 2024), where physical changes occur. Sleep and eating disturbances are strongly associated (Degasperi et al., 2024), particularly during adolescence (Cerolini et al., 2025). Previous research on adolescents and young adults has demonstrated that higher levels of body image dissatisfaction, which is common in girls with eating disorders, are significantly associated with sleep maintenance difficulties and non-restorative sleep (Soares et al., 2011; White et al., 2024). Moreover, less TIB is correlated with a greater desire for thinness, and this relationship is exacerbated by worse body dissatisfaction (White et al., 2024). There are potential pathways connecting sleep and disordered eating behaviour (for a review, see Cerolini, 2023). Heightened food reward sensitivity, greater emotional reactivity, reduced inhibitory control, and metabolic imbalances have been proposed as contributing factors that may increase the risk of higher energy intake in adolescents experiencing sleep difficulties (Duraccio et al., 2019; White et al., 2024). Moreover, strong longitudinal evidence in children and adolescents has connected sleep difficulties and disordered eating to a heightened risk of depression, anxiety, and other mental health issues (Cerolini et al., 2025). Given the established link between sleep and eating difficulties, alongside the increased risk of emotional problems during puberty and the greater prevalence of body and weight dissatisfaction among females (Mendle et al., 2020; Quittkat et al., 2019; Viner, 2015), these factors may contribute to the greater incidence of sleep difficulties in adolescent girls.

#### Social factors

Societal and environmental factors, such as stereotypes associated with educational issues and social expectations, may play a key role in explaining sex differences in psychological and sleep health during puberty (e.g., Baglioni & Palagini, 2022). Additionally, academic stress and attending school in an urban area seem to be negatively associated with sleep duration (L. N. Lin et al., 2017). It is possible that these factors impact differently girls’ and boys’ sleep duration and timing, or that an earlier delay in sleep timing in girls compared to boys makes them more vulnerable to sleep loss (L. N. Lin et al., 2017). Further studies on social factors affecting sleep health during puberty are needed to address this issue.

## Consequences of unhealthy sleep during puberty

Psychological and neuroscientific research has long indicated a close interaction between sleep and brain development in determining outcomes related to cognitive, emotional and behavioural development in adolescents (Baker & McMakin, 2024; J. Liu, Ji, et al., 2022). Reduced sleep quality and insufficient sleep tend to be associated with increased reactivity of the limbic system and heightened typical adolescent behaviours such as reward-seeking and risk-taking during this time (Baker & McMakin, 2024). Moreover, later sleep timing has also been associated with lower emotional, cognitive, and physical health indicators (e.g., Dutil et al., 2022).

The influence of sleep on health and psychophysical development appears to reach a critical moment during puberty. Research comparing adults and adolescents tends to report that the impact of sleep on emotional and relational well-being is stronger in adolescents, particularly during puberty (Baker & McMakin, 2024). Longitudinal studies also suggest that the association between internalizing symptoms and sleep disturbances is stronger in puberty than in childhood (Chai & Bian, 2024). During puberty, sleep is especially crucial for neuroaffective functioning for several reasons, including the heightened experience-dependent plasticity during sleep, making sleep pivotal for the remodelling of the adolescent brain in response to experiences (Baker & McMakin, 2024). In this regard, evidence indicate that good sleep efficiency may contribute to emotional wellbeing and modulate the impact of stress on sleep (Chiang et al., 2017). One putative mechanism for the effect of sleep on emotional wellbeing is that undisturbed REM sleep supports emotional memory processing, by allowing memories to be replayed in a low arousal environment (Baker & McMakin, 2024).

Unhealthy sleep appears to play a role in the development and maintenance of various forms of psychological distress and behavioural disorders. There are several lines of evidence that poor sleep not only affects adolescents’ psychological adjustment in the present and predisposes them to risks for future psychophysical problems, but also that previous sleep-related problems in childhood may increase the risk of exhibiting psychological symptoms during puberty (Lunsford-Avery et al., 2024).

### Physical health

Sleep appears to play an important role in promoting better physical health, although the causal relationships between sleep and measures of physical well-being are unclear. Several studies provide evidence for a relationship between insufficient sleep duration and poor sleep quality and an increased risk of obesity (Shochat et al., 2014) and impaired glucose metabolism (J. Liu, Ji, et al., 2022) in adolescents. In addition, some studies suggest an association between excessive daytime sleepiness and potentially harmful behaviors such as alcohol and energy drink consumption (Y. Liu et al., 2019), while adequate sleep is associated with healthier behaviours (Shochat et al., 2014). In general, sleep difficulties (and insomnia in particular) has been found to be a strong predictor of risky behaviors such as drug, alcohol and tobacco use, as well as risky injuries (Shochat et al., 2014). Longitudinal studies following girls and boys from early and middle adolescents to young adulthood showed that baseline shorter sleep duration and later sleep timing were associated with increased risk of alcohol and substance use (Hasler et al., 2017; Troxel et al., 2021).

Studies have shown that poor sleep is associated with early puberty (Gunawan et al., 2024; Puttawong et al., 2024). Some studies suggest that this association is mediated by increased risk of obesity (e.g., Tang et al., 2023). Although the exact mechanisms are debated, one putative explanation is that late bedtime and shortened sleep duration can alter the brain food reward system, encouraging overeating and unhealthy food consumption (Chaput, 2023). This pattern of consumption, not driven by metabolic need, increases risk of obesity, which in turn predispose to early pubertal development (Chaput, 2023). On the behavioral part, late bedtime is associated with more food consumption at night, with more high-sugar and processed foods consumed (Chaput, 2023; Dashti et al., 2015). Other processes may involve alterations of metabolic efficacy due to irregular and delayed sleep timing (Gonnissen et al., 2013; Ratwani et al., 2025).

Healthy sleep also contributes to youth’s physical fitness, as better sleep quality and adequate sleep duration are associated with better cardiorespiratory and muscle fitness in children and adolescents (Fonseca et al., 2021). Moreover, sleep duration has been found to predict the likelihood of being physically active, which is one of the most significant predictor of overall physical health (Grasaas et al., 2024).

In summary, good sleep health, including adequate sleep duration, good sleep efficiency and subjective quality, low daytime alertness, appear to be key aspects of physical health. However, most studies investigated the negative impact of unhealthy sleep, and more specifically of sleep difficulties, on different health indices, while the positive role of good sleep health needs more attention as a promotion target for overall physical health.

### Cognitive functioning

Pubertal development is a critical period for acquiring new skills and competence, including in the social and school context (Cheng et al., 2024). Complex cognitive functions, such as memory, attention, and planning, labelled as executive functions (EF), are crucial for daily functioning, mental health, social competence, and school performance (Esmaili et al., 2023).

A growing body of evidence indicates that sleep deprivation impairs memory consolidation and retrieval, attention, and problem solving (Balsamo et al., 2025; Chen et al., 2023; Kurinec et al., 2021; Newbury et al., 2021). Both in children and adolescents, insufficient sleep is associated with impairment in memory, attention and vigilance (J. Liu, Ji, et al., 2022; Mason & Saletin, 2023). Sleep insufficiency during puberty can thus have profound impact on adolescents’ ability to face daily challenges (Gruber et al., 2023).

Given these effects, it is not surprising that sleep duration appears to be the main dimensions associated with EF in adolescence (Gruber et al., 2023). As underscored by Richards et al. (2017), it should be noted that the association between sleep duration and cognitive performance across the lifespan is curvilinear, consistent with an optimal dose model of sleep: shorter and longer sleep durations are associated with impairments in cognitive functioning (Richards et al., 2017). In adults, the most studied cognitive function in association with sleep is memory, as sleep deprivation impairs the formation and retrieval of memories (e.g., R. Crowley et al., 2024). However, some studies on early and late adolescents (from 11 to 16 years of age) showed that verbal memory is not affected even by multiple nights of sleep deprivation, possibly due to neural compensation processes (Tarokh et al., 2016). Another key EF process is cognitive flexibility (CF), i.e. the ability to effectively adapt cognitive and behavioral strategies in response to changes in tasks or environmental demands (e.g., Diamond, 2013). CF is posited to be an important factors in adolescents’ daily functioning, as it allows for prospective changes and appropriate response to daily tasks (Sofia et al., 2020). One study found that after 24-h of sleep deprivation, late adolescents showed impairment in problem-solving, which were associated with impairment in CF (X. Zhang et al., 2024). De Bruin et al. (2017) reviewed studies on the effect of sleep manipulation on adolescents cognitive functioning, underlining that the most consistent finding is the negative effect of partial sleep restriction on psychomotor vigilance task. Moreover, studies showed that sleep extension and improvement lead to improved working memory (De Bruin et al., 2017). The effect of sleep on other aspects of cognitive functioning (e.g., memory consolidation) was more inconsistent, due to heterogeneity among studies; moreover, most studies did not account for the role of sleep quality in the effect of sleep manipulation of cognitive functioning (De Bruin et al., 2017).

Other dimensions of sleep health can also contribute to cognitive functioning and EF, with subsequent effects on school performance and other aspects of daily functioning. Perceived sleep quality is significantly associated with cognitive functions, with negative effects of poor sleep quality including impaired cognitive performance (Kuula et al., 2015), increased risk-taking behaviors associated with reduced cognitive control (Jex et al., 2025; Telzer et al., 2013), and reduced academic performance (Musshafen et al., 2021; Shochat et al., 2014).

Literature also underlies the importance of daytime alertness for cognitive performance and daily functioning in adolescence. For instance, a meta-analytic study including both children and adolescents (Dewald et al., 2010) found that sleepiness had the strongest relationship with academic performance, followed by sleep quality and sleep duration.

Sleep timing has also been investigated in association with executive functions in early adolescents, with most studies focused on circadian preference. For instance, a cross-sectional study on high school students found eveningness and daytime sleepiness were the best predictors of executive functioning (goal-planning and problem solving) than sleep duration (Cohen-Zion & Shiloh, 2018). Nonetheless, the effect of eveningness can be also imputed to the de-synchronisation between the timing of cognitive tasks and endogenous rhythms: performance in EF tasks is generally better at optimal times of day, which are later for evening types (Vidueira et al., 2023). Hahn et al. (2012) found a Chronotype × Time of Day interactions in cognitive functioning of early adolescents (11–14 years): when tested at their optimal time of day based on their circadian preference (morningness vs eveningness), adolescents performed better in EF tasks including inhibitory control, regardless of the amount of sleep the previous night (Hahn et al., 2012).

The distinct contribution of each dimension to cognitive performance in these age groups is not clear.

For instance, a meta-analysis of 11 studies performed in adolescents (Musshafen et al., 2021) found that overall academic performance was significantly associated with sleep quality but not sleep duration. However, as noted by Spruyt (2021), conclusions on the effects of sleep health on cognitive functions are limited by heterogeneity of sleep measures and concepts’ definition, cognitive assessment used, and type of study design. Moreover, studies differ in the potential confounder investigated, such as age, sex, comorbidities, stress, and familiar factors (Spruyt, 2021).

Overall, more evidence are needed to: investigate the complex relationship between different sleep health dimensions and cognitive functioning in early adolescence; deepen the positive role of sleep health in cognitive development and overall functioning in different life domains (e.g., academic, social).

### Internalizing symptoms

The literature seems to support a bidirectional association between sleep and internalizing disorders (Bacaro et al., 2024; Baker & McMakin, 2024; Chai & Bian, 2024; Xie et al., 2024). Several studies have investigated the other end of the continuum of sleep health, i.e. sleep disorders. Some studies identify problems in initiating and maintaining sleep during childhood as predictors of internalizing symptoms in early adolescents, particularly among females (J. P. L. Santos et al., 2025). For example, anxious adolescents may struggle to fall asleep as they tend to remain alert, while adolescents who struggle to get to sleep may indulge more frequently in repetitive negative thoughts that fuel anxiety and depressive symptoms (Blake et al., 2018; Hedin et al., 2020). Moreover, in early adolescence poor sleep may be associated with increased depressive symptoms through altered reward processing (Burani et al., 2021; Casement et al., 2016). Several studies report a reciprocal association between measures of sleep quality and mood (Uy & Gotlib, 2024; Xie et al., 2024), indicating how poor sleep quality tends to predict an increase in negative mood (Short et al., 2020; Xie et al., 2024) and depressive symptoms (Uy & Gotlib, 2024) in early adolescents. Furthermore, recent evidence suggests that sleep difficulties tend to mediate the relationship between exposure to stressful events and depressive symptoms during the transition from childhood to adolescence, particularly in females (Uy & Gotlib, 2024).

Due to the protective role of sleep health, emerging evidence indicates a bi-directional association between sleep quality and sleep duration and internalizing symptoms in adolescence (e.g., Bacaro et al., 2024). A recent meta-analysis of longitudinal studies showed the protective role of better sleep quality and longer sleep duration against internalizing symptoms in adolescence (Bacaro et al., 2024). On the other hand, measures of internalizing symptoms and psychological well-being predict youths’ sleep health (Bacaro et al., 2024). It is estimated that 9 h of sleep per night is required for optimal mood and that younger adolescents and those with higher levels of psychopathology need more sleep to have an optimal mood (Fuligni et al., 2019). The reasons for this may vary. For example, lack of sleep may promote negative, repetitive thoughts and reduce the subject’s ability to shift attention away from repetitive thoughts (Short et al., 2020). In addition, poor sleep can reduce REM sleep, which impairs the processing of emotional memories and thus increases arousal (Short et al., 2020). It should also be considered that not only does sleep tend to worsen mood in adolescents (Kearns et al., 2020), but that insufficient sleep is also associated with greater difficulty in regulating negative emotional states and greater reactivity to negative emotional stimuli (McMakin et al., 2016).

Several studies also suggest that sleep may be a protective factor for suicide risk in adolescence (Baldini et al., 2024; Chiu et al., 2018; Kearns et al., 2020), which is one of the leading causes of death in younger people (e.g., Longobardi et al., 2020). This association appears to be stronger in early adolescence than in later adolescence, and insomnia is the sleep disorder most strongly associated with suicidal ideation in adolescents (Longobardi et al., 2020). Adolescents with insomnia were almost three times more likely to attempt suicide and twice as likely to develop suicidal ideation compared to controls (Baldini et al., 2024). However, evidence indicate that short sleep duration and irregular sleep timing are also associated with suicidal ideation and behaviour in adolescents (Fernandes et al., 2021). For instance, it was estimated that 1-h increase in sleep duration led to a decreased risk of 11% of suicide plans in adolescents (Chiu et al., 2018). This is likely due to a reduction in serotonin production, which could increase impulsivity and thus the risk of carrying out a suicidal act. Moreover, both self-reported and actigraphy measured sleep variability longitudinally predict suicidal ideation and depressive symptoms in youths (Bernert et al., 2017).

### Externalizing symptoms

Most longitudinal studies available on this topic support a bidirectional relationship between sleep and externalizing symptoms, suggesting that the former tends to exacerbate the latter and vice versa (Liu, Magielski, et al., 2022). For instance, both sleep quality and sleep duration are protective against externalizing symptoms, which in turn can predict both sleep quality and duration in adolescents (Bacaro et al., 2024). Similarly, several lines of evidence suggest that poor sleep quality is associated with increased aggressive behaviours during the day in early adolescents (S. Lin, Fabris et al., 2024; Shimizu et al., 2020). In this sense, some activities found in young adolescents, such as excessive social media use during the night, could impair sleep quality and increase the risk of exhibiting aggressive behaviours in the following days, which also worsens the quality of social relationships and adjustment to the school environment (S. Lin, Fabris et al., 2024). In addition, the sleep health status of children and adolescents with neurodevelopmental disorders can impact the severity of their symptomatology (Bianca et al., 2024; Bruni et al., 2025). For instance, adolescents with Attention-Deficit/Hyperactivity Disorder (ADHD) tend to report more sleep disturbances than their healthy peers, and it is possible that such sleep disturbances exacerbate the emotional and behavioural difficulties of ADHD patients (Lunsford-Avery et al., 2016). In addition, early adolescents with ADHD who suffer from sleep disturbances appear to have poorer psychological adjustment than ADHD patients without sleep disturbances, reporting more symptoms and higher levels of depression (Marten et al., 2025). Specific dimensions, such as sleep quality, sleep duration and sleep-wake regularity, are particularly important for the overall functioning of children and adolescents with ADHD (Meneo et al., 2025).

### Stress and response to trauma

There is also evidence that adolescents with insufficient sleep duration or sleep problems tend to experience more stress (Kiss et al., 2024). This may be particularly relevant for adolescents exposed to social stressors or negative experiences, as some studies suggest that sleep disturbances longitudinally predict post-traumatic symptoms (Fan et al., 2017). On the other hand, adolescents who are exposed to particular social stressors or negative experiences at a young age tend to report more sleep disturbances, indicating a possible bidirectional relationship (Majeno et al., 2018). Good sleep health can have a positive impact on mental health in young populations exposed to stressful or traumatic experiences, potentially buffering against the development of post-traumatic symptoms (Coote et al., 2025). However, more longitudinal research is needed to model the relationship between sleep health and response to traumatic experiences in young adolescents (Coote et al., 2025).

### Psychosis

Few studies have examined sleep quality and sleep disturbance in relation to severe forms of psychopathology, such as psychotic disorders. However, there appears to be some evidence that sleep disturbances (including nightmares) play a role in the development and exacerbation of psychotic symptoms in adolescents at high risk of psychopathology (Lunsford-Avery et al., 2024).

Overall, puberty appears to be a critical period for the possible influences of sleep health on various areas of an individual’s psychophysical development. Further research is still needed to expand our knowledge of the influence of sleep health on adolescent well-being and to clarify which characteristics of sleep correlate with specific developmental outcomes, as well as the mechanisms through which they do so. However, the available data suggest that sleep is a central element in adolescent cognitive, emotional and behavioral development.

## Tailored interventions in early adolescence: where are we?

### Factors affecting the efficacy of sleep interventions in adolescents

The need for targeted interventions for sleep difficulties during adolescence is well-established. Indeed, adolescent sleep difficulties pose a risk for emotional and behavioural problems, and they tend to precede the development of anxiety, depression, and at-risk behaviours (de Zambotti et al., 2018; Lovato & Gradisar, 2014; McMakin & Alfano, 2015). Given the widespread problem, education school-based programs have been developed and tested, with sleep hygiene and unhelpful behaviours as main content (e.g., Gaskin et al., 2024). School-based programs from childhood to adolescence offer an opportunity to deliver cost-effective interventions to many youth in need (Gaskin et al., 2024). A recent systematic review highlights methodological issues in most studies, limiting the overall conclusion on their ineffectiveness and prompting for a more complex and multi-component approach to intervention (Gaskin et al., 2024). Specifically, these programs have proven to increase knowledge about sleep but not to consistently reduce sleep difficulties (Blake et al., 2019; Gaskin et al., 2024).

On the other hand of the sleep health continuum, sleep-wake disorders need specific interventions. Cognitive-Behavioural Therapy for Insomnia (CBT-I) is the first line treatment for the disorder, recommended for adults of any age and with different comorbidities (Riemann et al., 2023). The techniques included in standard CBT-I protocols include psychoeducation on sleep and insomnia, behavioural strategies such as stimulus control (i.e., to strengthen the association between the sleep environment and sleep) and sleep restriction (i.e., to increase sleep pressure), cognitive strategies (i.e., to reduce sleep-related worry and restructure dysfunctional beliefs about sleep), and arousal-targeting strategies (i.e., relaxation to reduce somatic and cognitive activation at bedtime) (Espie, 2022). Several randomized controlled trials (RCT) proved the effectiveness of cognitive-behavioural interventions in adolescents (e.g., Blake et al., 2019). Particularly effective are programs that incorporate CBT-I with interventions directed to circadian misalignment, such as light therapy or specific intervention modules, able to reduce both sleep difficulties and circadian disorders (Danielsson et al., 2016; Harvey et al., 2018).

Cognitive-behavioural interventions present some challenges, especially for a young population that presents widespread sleep health issues. A lack of CBT-I trained specialists and access barriers have been already discussed for adults (Baglioni et al., 2023; Espie, 2009). The same problems are present for delivering CBT-I and CBT-I-derived interventions to adolescents. Moreover, engaging adolescents is a key issue. Recently, these challenges have been summarized by Blake and colleagues (2019). The authors noted that cognitive-behavioural approaches to adolescent sleep problems need innovative technologies for a more personalised approach, greater scalability to reach a large population of adolescents, and flexible approaches to delivery (Blake et al., 2019). Specifically, while stepped-care approaches to sleep problems in adults have been extensively presented and discussed (Baglioni et al., 2023; Espie, 2009), there are no guidelines for adolescents.

Online approaches present different advantages in adolescence: they are scalable, deliverable to youth in remote areas, meet the need for autonomy, and can reduce the barrier represented by the uncertainty about entering a psychological treatment setting (Gradisar & Richardson, 2015). Digital CBT-I has been proven to be effective in reducing adolescent insomnia also in those with comorbid mental health problems (N. Bai & Yin, 2024; Cliffe et al., 2020). For instance, a recent RCT found that an adapted nurse-led digital CBT-I (dCBT-I) 6-week program on adolescents with depression and insomnia was effective at post-treatment and after 12 weeks (N. Bai & Yin, 2024). Although results are promising, a recent meta-analysis underlined the effectiveness in improving subjective perception of sleep but less robust results concerning some physiological measures (Cleary et al., 2025). The authors concluded that more high-quality RCTs comparing dCBT-I with face-to-face delivery in adolescents are needed.

Apart from the modality and the content of the intervention programs, the attitude with which they are planned and delivered is also relevant to achieve behavioural change in young populations (Yeager et al., 2018). As underlined by R. E. Dahl et al. (2018), it is crucial to develop programs based on student-driven and collaborative learning, as opposed to programs that inadvertently imply that adolescents require adults’ expertise and guidance to make the right behavioural choice.

### Adapting CBT-I to sleep health promotion

Some contents and theoretical principles of CBT-I can be adapted to promote sleep health in youths. Psychoeducation can target developmental sleep health changes, sleep hygiene practices most relevant to this age group (i.e., avoiding highly cognitively engaging activities at bedtime and limiting sleep-interfering substances), and the principles of sleep-wake regulation. It could be also important to illustrate the construct of sleep health as multidimensional, and that the quality, quantity, and timing of sleep are equally important for overall health. The theoretical ground of behavioural strategies can also be delivered, i.e. illustrating how spending awake time in bed and going to bed when not sleepy can lessen a positive association with sleep and alter the homeostatic pressure. A special attention should be paid in discussing daytime napping. This practice is frequent in adolescents trying to compensate for sleep insufficiency due to early school-start time coupled with later bedtime. While short daytime napping can increase daytime vigilance and cognitive performance in adolescents, long and mistimed naps push bedtime to later timing and increase sleep inertia, together with producing metabolic alterations (J. S. Santos et al., 2021). Cognitive techniques addressing worry and rumination can be beneficial for adolescents’ daily functioning and nighttime sleep, proposing ways to menage preoccupation and problems (i.e., through cognitive control). Worry specifically about sleep can be addressed with a mix of psychoeducation (i.e., how sleep is not under our control) and discussion of dysfunctional beliefs and attitudes about sleep. It is important to consider specific beliefs of adolescents over their sleep in this life period (Alvarado et al., 2024). Moreover, information on sleep health and, specifically, pubertal changes should also be given to parents/caregivers, addressing their specific beliefs about adolescents’ sleep (Robbins et al., 2022).

### Interventions targeting early adolescents

As reviewed above, pubertal development is marked by increased risk of unhealthy sleep due to changes at different levels. As a sensible period with rapid developmental changes, it can also be a unique period for behavioural changes, and thus the target of interventions to prevent sleep difficulties in adolescents and the cascade of events linking sleep and mental health (R. E. Dahl et al., 2018; M. E. Mitchell & Nugiel, 2024; Pfeifer & Allen, 2021). Moreover, interventions directed to change behaviours in the age group defined as early adolescence or late childhood (i.e., when pubertal development often occurs) can have increased efficacy compared to interventions directed to middle or late adolescence (Yeager et al., 2015, 2018). A review of interventions directed at achieving behavioural changes in youth, including nutrition and bullying, found that their efficacy is generally lower in middle and late adolescence compared to early adolescence (Yeager et al., 2018).

Few trials of interventions targeting early adolescents sleep, as compared to older adolescents, are available. A recent trial on peri-pubertal boys and girls with anxiety (ages 9–14) used a double approach to anxiety and sleep, as the two problems are known to reinforce reciprocally (McMakin et al., 2019). In a first step, participants underwent an anxiety treatment; in a second step, those who still had sleep issues were enrolled in a sleep treatment. Results showed that the anxiety treatment improved sleep without reaching clinical significance, with 75% of participants still above the cutoff, while the sleep treatment was effective in reducing sleep difficulties in peri-pubertal anxious participants (McMakin et al., 2019). The sleep intervention included 6–8 sessions of sleep enhancement based on CBT-I and using a motivational framework, targeting cognitive, emotional and behavioural processes at bedtime, personal motivation, development and maintenance of good sleep habits, sleep regularity and reduced media use at night, with a specific focus on anxiety and rumination at bedtime (R. Dahl et al., 2009; McMakin et al., 2019). Another trial on early adolescents (mean age 14.14 years) showed large improvements in sleep knowledge and smaller improvements in sleep quality and sleep hygiene, with those with lower sleep quality demonstrating larger improvements also in sleepiness (Illingworth et al., 2020). The program was school-based and aimed at increasing knowledge, facilitating sleep-related behavioural change and increasing motivation, using adaptations of CBT-I and stress management techniques. The program had a focus on positive attitudes toward sleep, self-reflection and personalization, addressing behavioural barriers to sleep and considering social influence (peer engagement in the program) (Illingworth et al., 2020).

Overall, innovative interventions to promote sleep health during adolescence can have positive effects on sleep habits, duration and quality, especially when they include specific techniques to manage cognitive, behavioural, and emotional factors influencing sleep. However, few studies have focused on puberty, although first evidence indicates that preventive and interventive approaches to sleep health during early adolescence can be effective in reducing sleep and psychological difficulties.

## Future directions

**Figure 2.**
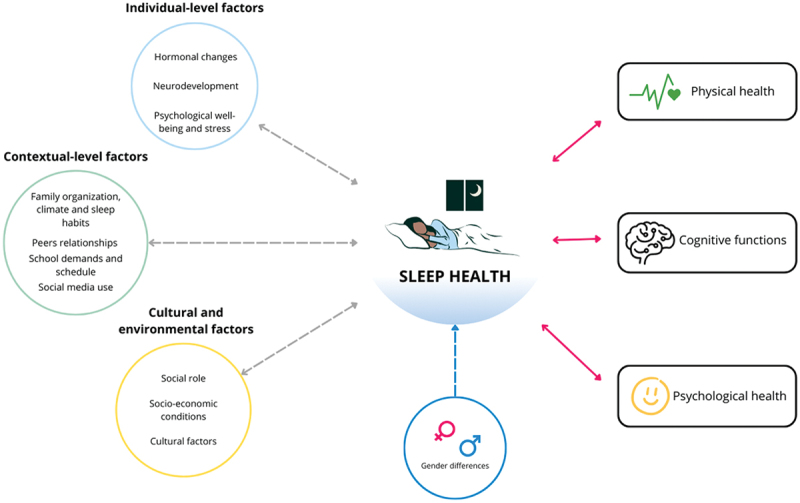
Influencing factors and outcomes of sleep health during pubertal development.

Puberty is a distinct period characterized by rapid multilevel changes influencing sleep health (see Figure 2 for an overview). Epidemiological and cross-sectional studies on sleep health and sleep difficulties in late childhood and adolescence often do not account for pubertal status (e.g., Gariepy et al., 2020). As opposed to other life stages in the paediatric population, puberty is not marked on chronological basis, as pubertal development starts and ends at different ages (Tanner, 1962). However, evidence underlines the need to consider pubertal status when investigating sleep health, its determinants, its consequences, and the interventive approaches used to promote sleep or reduce sleep difficulties (Lucien et al., 2021; Pfeifer & Allen, 2021). Among the factors affecting sleep health during pubertal development, biological sex and the socio-cultural construct around it (i.e., gender) emerge as critical in understanding trajectories of sleep health and psychological well-being from childhood to adulthood (Franco et al., 2020; Pengo et al., 2018). Literature on gender differences in sleep health, factors affecting sleep, and consequences of unhealthy sleep during puberty are relatively scarce. While most evidence focuses on hormonal aspects driving gender differences, less is known about socio-cultural differences (Franco et al., 2020). Future research could investigate aspects related to educational style, peer relationships, and parental involvement at bedtime in girls and boys to better understand how gender as a socio-cultural construct interacts with hormonal changes in shaping pubertal sleep health.

Dotted lines indicate influencing factors; unbroken lines indicate outcomes. The factors are posited as bidirectionally associated with sleep health. Gender differences are reported separately, as they influence sleep health also through the different influencing factors.

More studies are needed using the sleep health framework, specifically looking at: a) the distribution of sleep health dimensions during pubertal development; b) how gradations of sleep health dimensions are associated with individual-level, contextual-level, and cultural/environmental aspects of pubertal developments; c) the dimensions beyond sleep duration most associated with health outcomes.

From the literature, it emerges the unique opportunity offered by pubertal development to potentially prevent long-lasting psychological difficulties (e.g., Pfeifer & Allen, 2021). There is thus the need for more research on: a) tailored interventions considering pubertal changes at various levels influencing sleep; b) how improving sleep during pubertal development may influence the developmental trajectories of emotional and social adjustment during adolescence; c) experimentation with innovative technological mediums to deliver interventions to increase scalability; d) how to promote the translation of knowledge and attitudes fostered by school-based programs into individual behavioural improvements.

## Conclusions

Puberty is a pivotal and multifaceted developmental stage, marked by profound biopsychosocial changes that are significantly influenced by gender differences. These dynamic transformations during the pubertal period have a deep impact on an individual’s sleep health, with far-reaching consequences on physical and psychological health. A biopsychosocial approach to the unique challenges to sleep health during puberty is needed to modulate changes in sleep health during this developmental period.

To address the widespread challenges associated with sleep difficulties and related psychosocial issues during this critical stage, preventive and interventive approaches must be thoughtfully designed and implemented to target the unique needs and experiences of girls and boys navigating the pubertal transition. These comprehensive, evidence-based interventions can foster positive development during puberty and, later, adolescence.

## Data Availability

Data sharing does not apply to this article as no new data were created or analysed in this study.
